# National surveillance pilot study unveils a multicenter, clonal outbreak of VIM-2-producing *Pseudomonas aeruginosa* ST111 in the Netherlands between 2015 and 2017

**DOI:** 10.1038/s41598-021-00205-w

**Published:** 2021-10-25

**Authors:** Jannette Pirzadian, Marjolein C. Persoon, Juliëtte A. Severin, Corné H. W. Klaassen, Sabine C. de Greeff, Marcel G. Mennen, Annelot F. Schoffelen, Cornelia C. H. Wielders, Sandra Witteveen, Marga van Santen-Verheuvel, Leo M. Schouls, Margreet C. Vos, the Dutch CPE surveillance Study Group, L. Bode, A. Troelstra, D. W. Notermans, A. Maijer-Reuwer, M. A. Leversteijn-van Hall, J. A. J. W. Kluytmans, I. J. B. Spijkerman, K. van Dijk, T. Halaby, B. Zwart, B. M. W. Diederen, A. Voss, J. W. Dorigo-Zetsma, A. Ott, J. H. Oudbier, M. van der Vusse, A. L. M. Vlek, A. G. M. Buiting, S. Paltansing, P. de Man, A. J. van Griethuysen, M. den Reijer, M. van Trijp, E. P. M. van Elzakker, A. E. Muller, M. P. M. van der Linden, M. van Rijn, M. J. H. M. Wolfhagen, K. Waar, P. Schneeberger, W. Silvis, T. Schulin, M. Damen, S. Dinant, S. P. van Mens, D. C. Melles, J. W. T. Cohen Stuart, M. L. van Ogtrop, I. T. M. A. Overdevest, A. van Dam, H. Wertheim, H. M. E. Frénay, J. C. Sinnige, E. E. Mattsson, R. W. Bosboom, A. Stam, E. de Jong, N. Roescher, E. Heikens, R. Steingrover, E. Bathoorn, T. A. M. Trienekens, D. W. van Dam, E. I. G. B. de Brauwer, F. S. Stals

**Affiliations:** 1 Department of Medical Microbiology and Infectious Diseases, Erasmus MC University Medical Center Rotterdam, Rotterdam, The Netherlands; 2 Department of Medical Microbiology, University Medical Center Utrecht, Utrecht, The Netherlands; 3 Center for Infectious Diseases, Epidemiology and Surveillance, Center for Infectious Disease Control, National Institute for Public Health and the Environment (RIVM), Bilthoven, The Netherlands; 4 Center for Environmental Safety and Security, National Institute for Public Health and the Environment (RIVM), Bilthoven, The Netherlands; 5 Center for Infectious Diseases Research, Diagnostics and Laboratory Surveillance, Center for Infectious Disease Control, National Institute for Public Health and the Environment (RIVM), Bilthoven, The Netherlands; 6 Center for Infectious Disease Control, National Institute for Public Health and the Environment (RIVM), Bilthoven, The Netherlands; 7 Department of Medical Microbiology, ADRZ Medical Center, Goes, The Netherlands; 8 Department of Medical Microbiology, Alrijne Hospital, Leiden, The Netherlands; 9 Microvida Laboratory for Microbiology, Amphia Hospital, Breda, The Netherlands; 10 Department of Medical Microbiology, Amsterdam UMC (Location AMC), Amsterdam, The Netherlands; 11 Department of Medical Microbiology and Infection Control, Amsterdam UMC (Location VUmc), Amsterdam, The Netherlands; 12 Department of Medical Microbiology, Analytical Diagnostic Center N.V., Willemstad, Curaçao; 13 Department of Medical Microbiology, Atalmedial, Amsterdam, The Netherlands; 14 Department of Medical Microbiology, Bravis Hospital/ZorgSaam Hospital Zeeuws-Vlaanderen, Roosendaal/Terneuzen, The Netherlands; 15 Department of Medical Microbiology and Infectious Diseases, Canisius Wilhelmina Hospital, Nijmegen, The Netherlands; 16 Department of Medical Microbiology, CBSL, Hilversum, The Netherlands; 17 Department of Medical Microbiology, Certe, Groningen, The Netherlands; 18 Department of Medical Microbiology, Comicro, Hoorn, The Netherlands; 19 Department of Medical Microbiology, Deventer Hospital, Deventer, The Netherlands; 20 Department of Medical Microbiology and Immunology, Diakonessenhuis, Utrecht, The Netherlands; 21 Department of Medical Microbiology and Immunology, Elisabeth-TweeSteden Hospital, Tilburg, The Netherlands; 22 Microbiology and Infection Prevention, Franciscus Gasthuis and Vlietland, Schiedam, The Netherlands; 23 Department of Medical Microbiology, Gelderse Vallei Hospital, Ede, The Netherlands; 24 Department of Medical Microbiology and Infection Prevention, Gelre Hospitals, Apeldoorn, The Netherlands; 25 Department of Medical Microbiology and Infection Prevention, Groene Hart Hospital, Gouda, The Netherlands; 26 Department of Medical Microbiology, Haga Hospital, ‘s-Gravenhage, The Netherlands; 27 Department of Medical Microbiology, HMC Westeinde Hospital, ‘s-Gravenhage, The Netherlands; 28 Department of Medical Microbiology, IJsselland Hospital, Capelle a/d IJssel, The Netherlands; 29 Department of Medical Microbiology, Ikazia Hospital, Rotterdam, The Netherlands; 30 Laboratory of Medical Microbiology and Infectious Diseases, Isala Hospital, Zwolle, The Netherlands; 31 Department of Medical Microbiology, Izore Center for Infectious Diseases Friesland, Leeuwarden, The Netherlands; 32 Department of Medical Microbiology and Infection Control, Jeroen Bosch Hospital, ‘s-Hertogenbosch, The Netherlands; 33 LabMicTA, Regional Laboratory of Microbiology Twente Achterhoek, Hengelo, The Netherlands; 34 Department of Medical Microbiology, Laurentius Hospital, Roermond, The Netherlands; 35 Department of Medical Microbiology, Maasstad Hospital, Rotterdam, The Netherlands; 36 Department of Medical Microbiology, Maastricht University Medical Center, Maastricht, The Netherlands; 37 Department of Medical Microbiology, Meander Medical Center, Amersfoort, The Netherlands; 38 Department of Medical Microbiology, Noordwest Ziekenhuisgroep, Alkmaar, The Netherlands; 39 Department of Medical Microbiology, Onze Lieve Vrouwe Gasthuis, Amsterdam, The Netherlands; 40 Department of Medical Microbiology, PAMM, Veldhoven, The Netherlands; 41 Public Health Laboratory, Public Health Service, Amsterdam, The Netherlands; 42 Department of Medical Microbiology, Radboud University Medical Center, Nijmegen, The Netherlands; 43 Department of Medical Microbiology, Regional Laboratory Medical Microbiology, Dordrecht, The Netherlands; 44 Department of Medical Microbiology, Regional Laboratory of Public Health, Haarlem, The Netherlands; 45 Department of Medical Microbiology, Reinier de Graaf Groep, Delft, The Netherlands; 46 Laboratory for Medical Microbiology and Immunology, Rijnstate Hospital, Velp, The Netherlands; 47 Department of Medical Microbiology, Saltro Diagnostic Center, Utrecht, The Netherlands; 48 Department of Medical Microbiology, Slingeland Hospital, Doetinchem, The Netherlands; 49 Department of Medical Microbiology and Immunology, St. Antonius Hospital, Nieuwegein, The Netherlands; 50 Department of Medical Microbiology, St. Jansdal Hospital, Harderwijk, The Netherlands; 51 Department of Medical Microbiology, St. Maarten Laboratory Services N.V., Cay Hill, St. Maarten; 52 Department of Medical Microbiology, University Medical Center Groningen, Groningen, The Netherlands; 53 Department of Medical Microbiology, VieCuri Medical Center, Venlo, The Netherlands; 54 Department of Medical Microbiology and Infection Control, Zuyderland Medical Center, Sittard-Geleen, The Netherlands; 55 Department of Medical Microbiology and Infection Control, Zuyderland Medical Center, Heerlen, The Netherlands

**Keywords:** Bacterial infection, Clinical microbiology, Antimicrobial resistance, Next-generation sequencing

## Abstract

Verona Integron-encoded Metallo-beta-lactamase (VIM) is the most frequently-encountered carbapenemase in the healthcare-related pathogen *Pseudomonas aeruginosa*. In the Netherlands, a low-endemic country for antibiotic-resistant bacteria, no national surveillance data on the prevalence of carbapenemase-producing *P. aeruginosa* (CPPA) was available. Therefore, in 2016, a national surveillance pilot study was initiated to investigate the occurrence, molecular epidemiology, genetic characterization, and resistomes of CPPA among *P. aeruginosa* isolates submitted by medical microbiology laboratories (MMLs) throughout the country. From 1221 isolates included in the study, 124 (10%) produced carbapenemase (CIM-positive); of these, the majority (95, 77%) were positive for the *bla*_VIM_ gene using PCR. Sequencing was performed on 112 CIM-positive and 56 CIM-negative isolates (n = 168), and genetic clustering revealed that 75/168 (45%) isolates were highly similar. This genetic cluster, designated Group 1, comprised isolates that belonged to high-risk sequence type ST111/serotype O12, had similar resistomes, and all but two carried the *bla*_VIM-2_ allele on an identical class 1 integron. Additionally, Group 1 isolates originated from around the country (i.e. seven provinces) and from multiple MMLs. In conclusion, the Netherlands had experienced a nationwide, inter-institutional, clonal outbreak of VIM-2-producing *P. aeruginosa* for at least three years, which this pilot study was crucial in identifying. A structured, national surveillance program is strongly advised to monitor the spread of Group 1 CPPA, to identify emerging clones/carbapenemase genes, and to detect transmission in and especially between hospitals in order to control current and future outbreaks.

## Introduction

Carbapenemase-producing *Pseudomonas aeruginosa* (CPPA) is an emerging pathogen responsible for many serious healthcare-related infections worldwide^1,2^ Carbapenemases hydrolyze important antibiotics for treating *P. aeruginosa* infections, and frequently co-occur with other resistance mechanisms, resulting in a multidrug-resistant (MDR) or extensively drug-resistant (XDR) phenotype^1,3,4^. In 2017, the World Health Organization prioritized carbapenem-resistant *P. aeruginosa* among the top three antibiotic-resistant bacteria in “critical” need of new antibiotics^5^. Verona Integron-encoded Metallo-beta-lactamase (VIM) is the most frequently-encountered carbapenemase in *P. aeruginosa*^3^. Among variants, the VIM-2 metallo-beta-lactamase exhibits the broadest geographical distribution, including on the European continent^6,7^.

In the Netherlands, there is low endemicity for antibiotic-resistant bacteria^8^. In 2018, only 2% of Dutch clinical *P. aeruginosa* isolates were MDR (i.e. resistant to ≥ 3 antimicrobial groups), but among MDR isolates, around 50% were phenotypically resistant to carbapenems^8^. The first Dutch CPPA outbreak was reported in 2011 by one tertiary-care hospital; an investigation into isolates obtained between 2008 and 2009 that were resistant to imipenem revealed that 33% contained the *bla*_VIM_ gene, and most belonged to a single genetic cluster^9^. A surveillance study in 2012 based on convenience sampling, incorporating isolates from 2009 to 2011 and 21 medical microbiology laboratories (MMLs), similarly unveiled one large genetic cluster of VIM-2-producing *P. aeruginosa* belonging to sequence type ST111/serotype O12^10^. This high-risk, epidemic lineage is one of the most globally-widespread MDR/XDR *P. aeruginosa* lineages, and is associated with high morbidity and mortality in patients^6,10,11^. Since then, ST111/O12 has continued to be isolated from patients and hospital environments, and based on sporadic studies, appeared to be the most frequently-encountered CPPA sequence type in the Netherlands^10,12,13^. The outcomes of these studies also suggested that CPPA may be distributed nationwide in the Netherlands, but systematically-collected, national surveillance data was not available.

In 2016, a national surveillance pilot study was initiated by the National Institute for Public Health and the Environment (RIVM) in the Netherlands to determine the necessity of a structured surveillance program for CPPA. This was in addition to an existing surveillance program on carbapenemase-producing *Enterobacterales* that began in 2011; during that time, the RIVM had also coincidentally received *P. aeruginosa* isolates from several participating MMLs. In this study, *P. aeruginosa* isolates collected by the RIVM between 2015 and 2017 were analyzed to estimate the occurrence of CPPA among submitted isolates, characterize their genetic environment and molecular epidemiology using a whole-genome multilocus sequence typing (wgMLST) scheme, and determine their antibiotic resistance gene profiles.

## Results

### CPPA occurrence among submitted isolates

From January 2015 until December 2017, 39 Dutch MMLs submitted 1445 confirmed *P. aeruginosa* isolates with reduced sensitivity to meropenem and/or imipenem. These isolates were subjected to the carbapenemase inactivation method (CIM) to assess possible carbapenemase production. Only the first carbapenemase-producing (CIM-positive) and first non-carbapenemase-producing (CIM-negative) isolate per patient submitted during the study period were included, resulting in a total of 1221 isolates from 1216 patients, five of whom carried both a CIM-positive and CIM-negative *P. aeruginosa* isolate. For NGS, only a single isolate per patient was used.

Carbapenemase production was observed in 124 (10%) isolates using the CIM test; of these, 107 (86%) isolates were also positive for a carbapenemase gene using PCR (Table 1). All 1097 non-carbapenemase-producing isolates were also carbapenemase-PCR negative. The majority of CIM-positive isolates (77%, 95/124) carried a gene belonging to the *bla*_VIM_ family. Analysis of next-generation sequencing (NGS) data on 83 CIM-positive, *bla*_VIM_-PCR-positive isolates revealed that they all carried the *bla*_VIM-2_ allele. Nine isolates carried a *bla*_IMP_ gene comprising five different *bla*_IMP_ alleles. Three isolates carried a *bla*_NDM-1_ allele. Seventeen CIM-positive isolates did not yield a PCR product, and were sequenced; three carried a *bla*_GES-5_ allele, but a carbapenemase-encoding gene could not be found in the remaining 14 isolates.

**Table 1:** Distribution of carbapenemase genes in CIM-positive *P. aeruginosa* isolates collected within the Netherlands between 2015 and 2017.

Carbapenemase PCR	Sampling year			Total	Carbapenemase allele	Sampling year				Total
	2015	2016	2017			2015	2016		2017	
**CIM-positive**					*bla*_VIM-2_	19	16		48	83
*bla*_VIM_	31	16	48	95	*bla*_IMP-13_	2	1			3
*bla*_IMP_	3	2	4	9	*bla*_IMP-1_				2	2
*bla*_NDM_		1	2	3	*bla*_IMP-7_	1			1	2
PCR negative	4	8	5	17	*bla*_IMP-8_				1	1
Total	38	27	59	124	*bla*_IMP-26_		1			1
**CIM-negative**					*bla*_NDM-1_		1		2	3
PCR negative	311	372	414	1097	*bla*_GES-5_	1			2	3
**Total**	349	399	473	1221	No carba gene found	3	8		3	14
					**Total**	26	27		59	112

In the left table, the results from CIM tests and multiplex PCRs for all isolates included in the study are shown. From 1221 *P. aeruginosa* isolates that were included, 124 (10%) were CIM-positive. In 107 CIM-positive isolates, a carbapenemase gene was also detected. All *bla*_KPC_ and *bla*_OXA-48_ PCRs were negative, and are therefore not shown. In the right table, NGS results are shown. From the 124 CIM-positive isolates, 112 were sequenced, and the *bla*_VIM-2_ allele was discovered in 83 (74%) isolates.

*VIM* Verona Integron-encoded Metallo-beta-lactamase, *IMP* Imipenem Metallo-beta-lactamase, *NDM* New Delhi Metallo-beta-lactamase, *GES* Guiana Extended-Spectrum beta-lactamase, *Carba* carbapenemase.

### Sequencing revealed a large genetic cluster of bla_VIM-2_-containing CPPA

NGS was performed on 112 CIM-positive isolates, and on 56 CIM-negative isolates that were matched to CIM-positive isolates based on MML and sampling year. Demographic data provided by MMLs on the patients from which these isolates derived is available in Supplementary Table S1. In some regions, *P. aeruginosa* was not submitted or found, so isolates from these regions were not sequenced. A complete geographical overview of CIM-positive and CIM-negative isolates that were included is available in Supplementary Figure S1.

**Figure 1:**
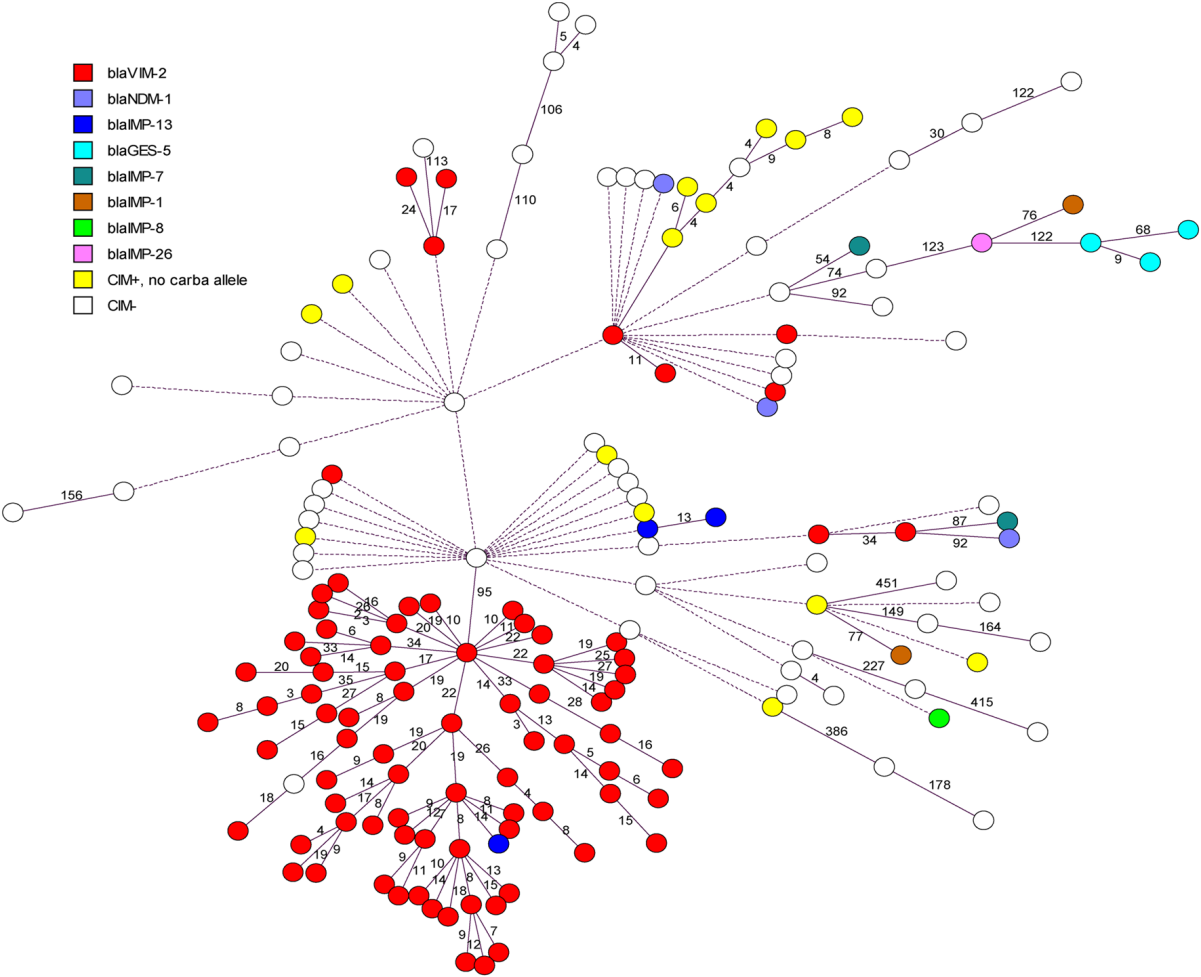
Minimum-spanning tree of 112 CIM-positive *P. aeruginosa* isolates and 56 CIM-negative *P. aeruginosa* isolates based on wgMLST analysis. Each circle represents an isolate. Colors indicate the carbapenemase-encoding gene. Yellow circles (CIM+, no carba allele) indicate CIM-positive isolates in which no carbapenemase-encoding gene could be identified. White circles indicate CIM-negative isolates. Numbers on the lines between circles indicate the number of allelic differences between isolates. To avoid long branches, lines connecting circles are logarithmic representations of allelic distances. For allelic distances larger than 3500 genes, a dashed line is used and distances are not shown. All minimum spanning trees were created in BioNumerics v7.6, exported as metafiles, and imported into Adobe Illustrator (Adobe Creative Cloud 2020, www.adobe.com).

Genotypic relationships between isolates were determined using wgMLST (Fig. 1). There was a high degree of genotypic diversity, with large allelic distances often exceeding > 3500 alleles between isolates. However, 75/168 (45%) isolates partitioned closely together with a maximum distance of 35 alleles. This group, designated Group 1, comprised isolates that were all CIM-positive (with the exception of one isolate), all belonged to sequence type ST111 and serotype O12, and all but two isolates carried the *bla*_VIM-2_ allele; one isolate carried *bla*_IMP-13_, and in the other, a CIM-negative isolate, no carbapenemase-encoding gene could be identified.

**Figure 2:**
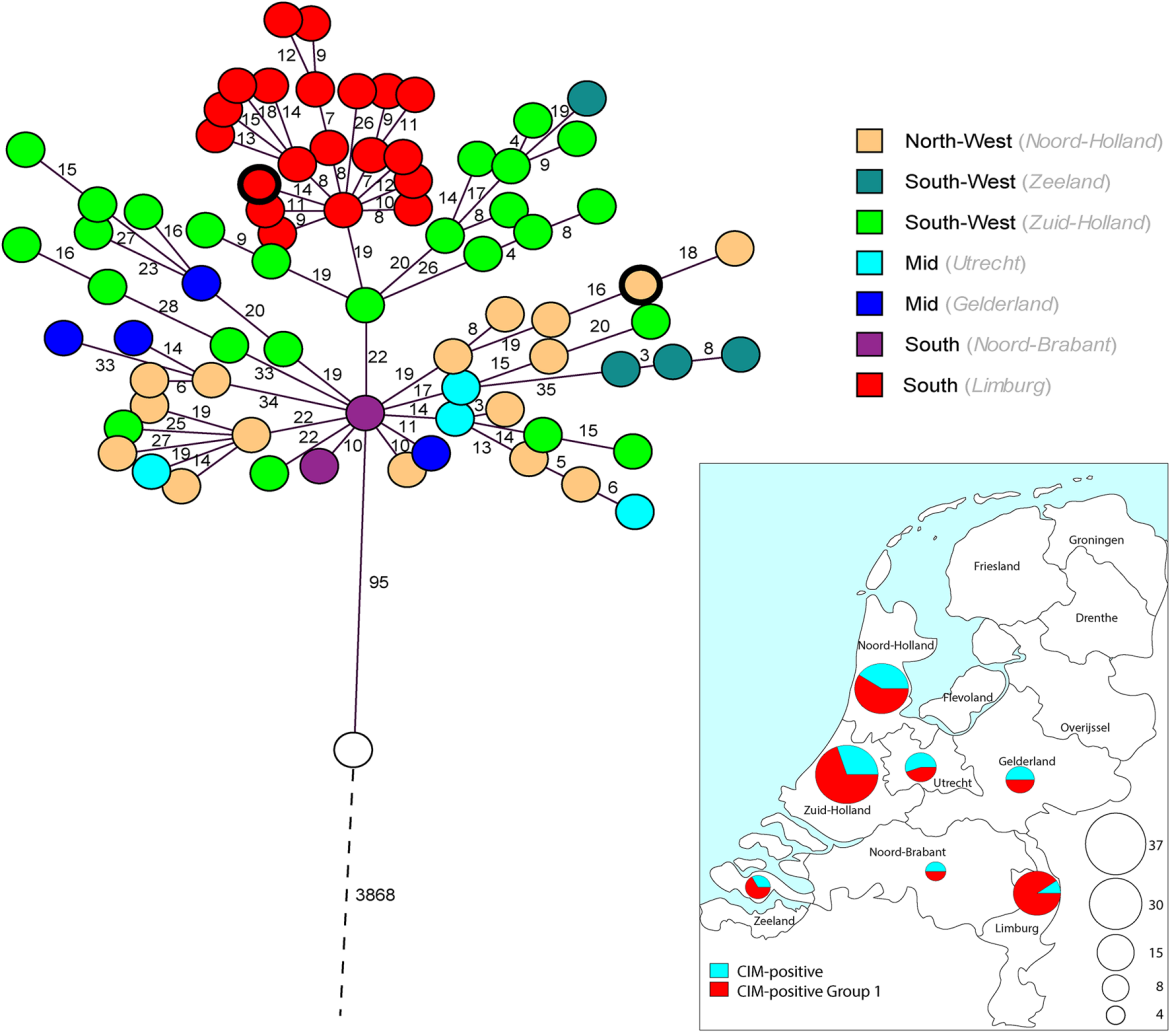
Geographical distribution of Group 1 isolates in the Netherlands. Above, Minimum-spanning tree of Group 1 isolates based on wgMLST analysis. Each circle represents an isolate. Colors indicate the region where the isolate was obtained. Dutch provinces are given in italics. The red circle with a thick border represents the CIM-positive Group 1 isolate that carried a *bla*_IMP-13_ gene. The beige circle with a thick border represents the CIM-negative Group 1 isolate. The white circle represents the CIM-negative ST111/O12 isolate most closely related to Group 1. For understanding distances between isolates, refer to Fig. 1. Below, Map of the Netherlands showing the distribution of 112 CIM-positive *P. aeruginosa* isolates that were sequenced in this study, and the number of those isolates that were Group 1, by province. Map was created by importing a screen capture into Adobe Illustrator 2020 and plotting the origin of isolates using the Type-Ned MRSA website (www.typened-mrsa.rivm.nl).

Within Group 1, several genetic clusters could be seen with few allelic differences between isolates. One CIM-negative isolate (also belonging to ST111/O12) was separated from Group 1 by only 95 allelic differences. All other isolates differed from Group 1 by > 3500 allelic differences. Group 1 also comprised isolates from seven Dutch provinces (Fig. 2).

**Figure 3:**
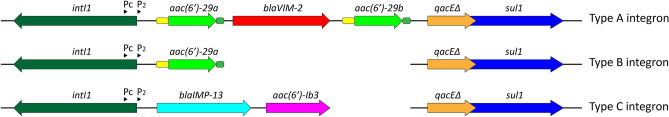
Class 1 integron compositions of Group 1 isolates. All Group 1 isolates except two carried a 3554 bp Type A integron containing the *bla*_VIM-2_ gene cassette flanked by *aac(6′)-29a* and *aac(6′)-29b* genes. Yellow and green squares flanking the *aac(6′)-29a/b* genes denote identical sequences. The integron structure of the CIM-negative Group 1 isolate was identical to a Type A integron, but the *bla*_VIM-2_ and *aac(6′)-29b* genes had been deleted (Type B). The IMP-13-encoding Group 1 isolate carried a different gene cassette composition (Type C). *P*, promoter located within the integrase gene (*intI1*); *aac(6′)-29a*, *aac(6′)-29b*, and *aac(6′)-Ib3*, aminoglycoside resistance genes; *bla*_VIM-2_ and *bla*_IMP-13_, carbapenem resistance genes; *qacEΔ,* incomplete quaternary ammonium compound resistance gene; *sul1*, sulfonamide resistance gene. Figure was created using BioNumerics v7.6 and Adobe Illustrator 2020.

The composition of the integron regions of six Group 1 isolates was reconstructed by combined short- and long-read sequencing. This showed that all four VIM-2-encoding Group 1 isolates contained an identical class 1 integron (Type A) carrying the *intI* integrase gene, the *bla*_VIM-2_ gene flanked by the aminoglycoside resistance genes *aac(6′)-29a* and *aac(6′)-29b*, an incomplete *qacE* gene involved in quaternary ammonium compound resistance, and the sulfonamide resistance gene *sul1* (Fig. 3). In the CIM-negative Group 1 isolate, the *bla*_VIM-2_ and *aac(6′)-29b* genes were lost from this integron by deletion (Type B). The IMP-13-encoding Group 1 isolate was also shown to carry an integron (Type C) similar to the Type A integron, but contained the *bla*_IMP-13_ gene and the aminoglycoside resistance gene *aac(6′)-Ib3*. Mapping the Illumina reads of the other VIM-2-encoding Group 1 isolates against these reconstructed integrons showed that all carried the Type A integron.

### Group 1 isolates were distinct from internationally-derived CPPA isolates

**Figure 4:**
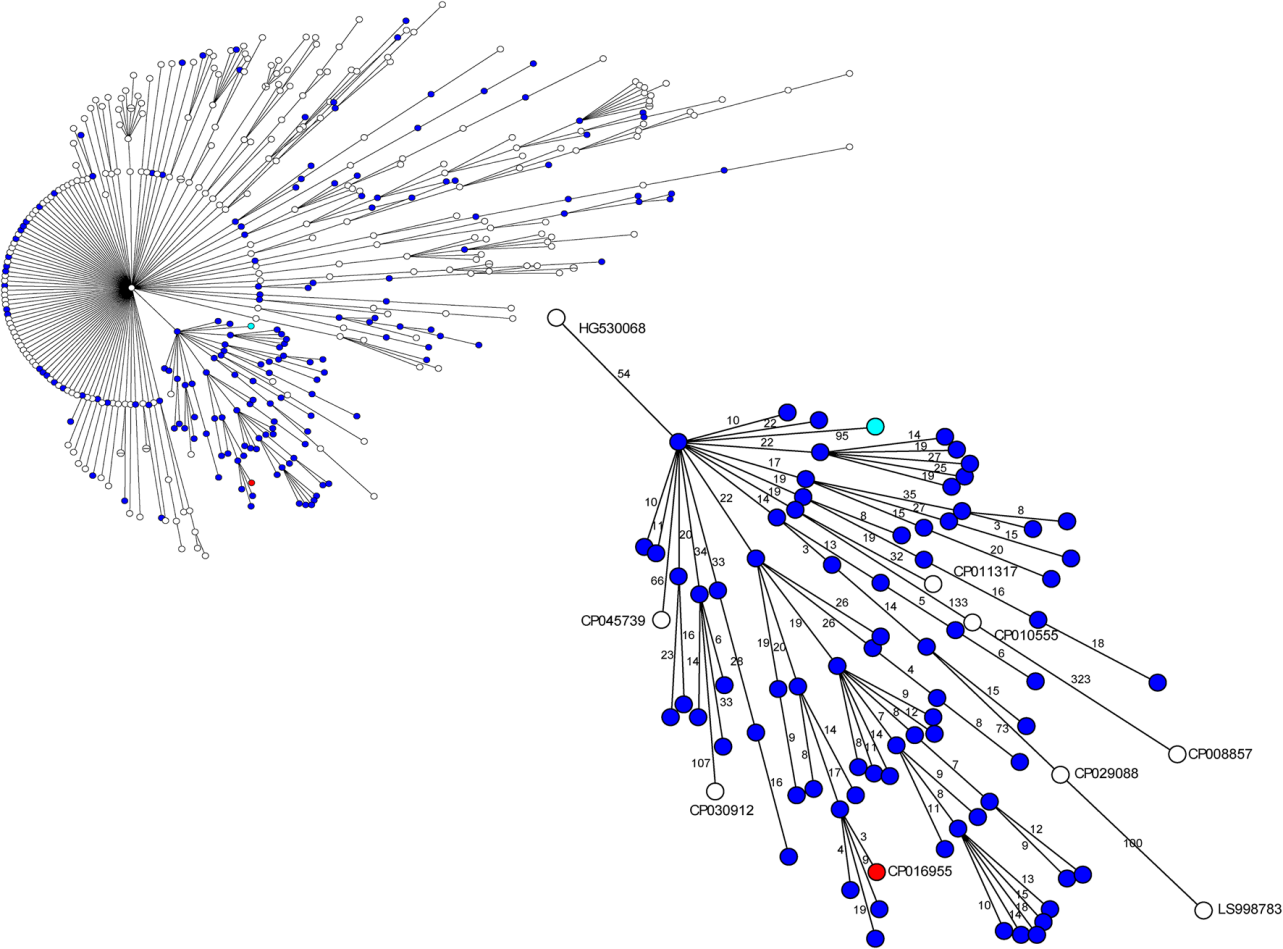
Minimum-spanning tree of the 168 *P. aeruginosa* isolates sequenced in this study (blue circles), and of 260 *P. aeruginosa* chromosomal sequences obtained from the NCBI GenBank database (white circles) based on wgMLST analysis. The enlarged portion of the tree displays Group 1 isolates, and the closely-related NCBI sequences with their accession numbers. The teal circle denotes the CIM-negative ST111/O12 isolate from this study that partitioned the closest to Group 1. The red circle indicates the Erasmus MC reference isolate that was used to design the wgMLST scheme. For understanding distances between isolates, refer to Fig. 1.

The wgMLST profiles of the 168 sequenced isolates in this study were compared to 260 complete, annotated *P. aeruginosa* chromosomal sequences from the National Center for Biotechnology Information’s GenBank database (Fig. 4). Group 1 is shown in the zoomed in panel; notably, the isolates sequenced in this study (blue circles) were interconnected and not interrupted by a different *P. aeruginosa* sequence (white circles). However, four isolates with the *bla*_VIM-2_ gene were closely related to Group 1, separated by only 3–66 allelic differences. The first strain (accession no. CP016955) was RIVM-EMC4982 from the Erasmus MC University Medical Center Rotterdam, which was the reference isolate used to design the wgMLST scheme for this study. The second strain, Carb01 63 (accession no. CP011317), originated from Maasstad Hospital, another hospital in Rotterdam, the Netherlands. The third strain, PA38182 (accession no. HG530068), originated from a hospital in the United Kingdom, and has been involved in several major outbreaks^14,15^. The fourth strain, PaeAG1 (accession no. CP045739), was a MDR strain isolated from a patient with pneumonia admitted to intensive care in Costa Rica, and was the first strain reported to carry two carbapenemase-encoding genes (*bla*_VIM-2_ and *bla*_IMP-18_)^16^. Five other GenBank isolates partitioned with Group 1 at larger distances (separated by 77–400 allelic differences). These isolates did not carry the *bla*_VIM-2_ gene, but one (accession no. LS998783) carried the carbapenemase-encoding gene *bla*_GES-5_.

### Antibiotic resistance gene profiles and QRDR analysis

ResFinder analyses showed that all 168 *P. aeruginosa* sequenced isolates carried the beta-lactamase gene *bla*_PAO_, the aminoglycoside resistance gene *aph(3′)-IIb*, and the fosfomycin resistance gene *fosA* (Supplementary Table S2). All but four isolates carried the chloramphenicol transferase gene *catB7*. Twenty-six other genes encoding beta-lactamase production, and 27 other genes associated with aminoglycoside resistance, were also found among the 168 isolates. The most prevalent beta-lactamase gene (56%, 94/168) was the *bla*_OXA-50_-like gene *bla*_OXA-395_. This gene was present in all Group 1 isolates, in 29% (11/38) of CIM-positive isolates that did not belong to Group 1, and in 15% (8/55) of CIM-negative isolates that did not belong to Group 1. Both *aac(6')-29a* and *aac(6′)-29b* genes were found in all 73 VIM-2-encoding Group 1 isolates; only *aac(6')-29a* was present in the CIM-negative Group 1 isolate, and neither gene was present in the IMP-13-encoding Group 1 isolate, confirming the integron compositions of Group 1 isolates after read-mapping. Among other isolates, one of either gene was found in three of the 38 CIM-positive isolates that did not belong to Group 1, but neither gene was found in any CIM-negative isolate that did not belong to Group 1.

An analysis of quinolone resistance-determining regions (QRDR) for genes *gyrA*, *gyrB*, *parC*, and *parE* in all sequenced isolates revealed that the *gyrA* T83I and the *parC* S87L mutations were found in 100% of ST111 isolates, but were also common among other sequence types; therefore, no unique mutation pattern could be determined for Group 1 isolates (Supplementary Table S3). The combination of T83I and D87N mutations in *gyrA* were only found in 60% (3/5) of ST175 isolates. The S87W mutation in *parC* was exclusively found in ST175 isolates.

## Discussion

This national surveillance pilot study unveiled that the Netherlands had experienced an ongoing, nationwide, inter-institutional outbreak of a single, clonal genetic cluster of VIM-2-producing *P. aeruginosa* over a period of at least three years. It was clear from previous reports that several individual hospitals had already recognized this outbreak within their own settings. After the single-center CPPA outbreak reported by van der Bij et al*.*, a surveillance study into 11 hospitals in the Netherlands in 2012 found that the outbreak by CPPA belonging to ST111 was widespread^10^. The surprising results of that study, however, were not followed by the implementation of a structured, national surveillance program. The current study revealed that ST111/O12 has continued to prevail in the Netherlands, and that the outbreak has involved multiple MMLs distributed over a large part of the country.

Genetic clustering of 168 sequenced isolates revealed that almost half (45%, 75/168) showed high genetic similarity, and, with the exception of two isolates, carried the *bla*_VIM-2_ allele. Few differences (≤ 35 alleles) were seen between isolates in this genetic cluster, designated Group 1. All VIM-2-encoding Group 1 isolates possessed identical integron structures; coupled with the fact that Group 1 isolates originated from around the country and were submitted by multiple MMLs, we could confirm that Group 1 CPPA have clonally spread throughout the Netherlands. Notably, Group 1 isolates were genetically distinct compared to the other sequenced isolates, exceeding 3500 allelic differences, and to most publicly-available sequences on GenBank. As *P. aeruginosa* strain Carb01 63 originated from the Netherlands and was isolated in 2012, the authors suspect that this strain also belonged to the outbreak described by this study. Like Carb01 63, Group 1 isolates belonged to sequence type ST111/O12, the predominant *P. aeruginosa* lineage in Europe^6,7,10^.

*P. aeruginosa* ST111/O12 clones exhibit high morbidity and mortality in infected patients^6,11,15^. ST111/O12 was first reported in the Netherlands in 2003, more than a decade before the inclusion period of this study, and then again in 2005^9,13^. It is reasonable to suspect that the inter-institutional outbreak described by this study, and the multicenter outbreak described in 2012 by van der Bij et al., are linked, and may have started much earlier than previously anticipated. It is unclear when ST111/O12 was first introduced to the Netherlands, or how country-wide transmission could have occurred. Since it is known that patient referral networks can contribute to the spread of high-risk clones within a country, the transfer of patients between Dutch healthcare institutions most likely played a role in transmission, especially in cases of unnoticed colonization^17^. To date, there have been no studies analyzing the impact of patient transfers between healthcare institutions in the Netherlands. It is highly recommended that patient transfers include accompanying reports on colonization by highly-resistant microorganisms to limit potential inter-institutional transmission. In case of increasing prevalence rates encountered via a national surveillance system, an additional measure could be a national policy to screen patients for CPPA on admission. Screening should especially be performed in patients with a history of recent hospitalization in another healthcare center reporting a CPPA outbreak. Furthermore, CPPA, including ST111/O12 clones, have been shown to reside in the wet niches of hospitals through the formation of biofilm reservoirs^12,14,18^. These reservoirs are persistent, may resist disinfection, and can disperse CPPA to vulnerable patient populations^19,20^, so identifying and limiting environmental sources of ST111/O12 clones in hospitals are of particular interest. ST111/O12 clones have also been found outside of hospitals in wastewater effluents^21,22^; as the presence of CPPA reservoirs outside of healthcare institutions was not investigated during this study, community-acquired CPPA infections cannot be ruled out.

This study has some limitations. Firstly, no epidemiological data were collected, so no risk factors could be conclusively linked to the outbreak, and transmission events could not be identified. Secondly, MML compliance with submitting isolates was not checked. Thirdly, not all CPPA could be sequenced with the Illumina platform due to budgetary reasons, so it is possible that additional emerging genetic clusters had been missed among the other included isolates; selection bias was limited by sequencing all CPPA isolates received between 2016 and 2017, and sequencing a random selection of CPPA isolates that had been voluntarily submitted in 2015. Finally, long-read sequencing was only performed for six Group 1 isolates.

As a result of this national surveillance pilot study, MMLs have been advised to continue submitting *P. aeruginosa* isolates to the RIVM for NGS and surveillance. Only isolates with demonstrable carbapenemase production and/or a carbapenemase-encoding gene(s) have been requested. However, structured, national surveillance is still lacking in the Netherlands, since results from current CPPA surveillance are only reported in the Type-Ned database. National surveillance should include alerting the MMLs involved, and supporting epidemiological investigations into possible transmission routes; this is especially important for new, emerging clones, such as a clone with *bla*_GES-5_. National surveillance should also collect epidemiological data so that risk factors can be assessed. Additionally, the RIVM developed an in-house wgMLST scheme for *P. aeruginosa* to investigate the clonality of submitted isolates for this study, but recently several other validated schemes were published that may aid surveillance and outbreak investigations^23–25^.

In conclusion, the widespread distribution of Group 1 CPPA throughout the Netherlands went unnoticed for a period of at least three years, and this national surveillance pilot study was crucial in identifying the outbreak. Based on previous reports, it is likely that this inter-institutional outbreak started even earlier than previously anticipated. Therefore, the authors strongly recommend the implementation of a structured, national surveillance program in the Netherlands that incorporates wgMLST to monitor the spread of Group 1 CPPA, to identify emerging clones/carbapenemase genes, and to detect transmission in and especially between hospitals to control current and future outbreaks.

## Methods

### Inclusion of isolates

In 2016, all Dutch MMLs were sent a letter requesting that *P. aeruginosa* isolates be sent to the RIVM for a national surveillance pilot study. Criteria for submission were that isolates had a minimum inhibitory concentration of > 2 µg/ml for meropenem or > 4 µg/ml for imipenem (as determined by the MML’s preferred antimicrobial susceptibility method), and that one isolate per-person-per-year-per-lab was submitted. For each submitted isolate, MMLs were also requested to provide patient age, patient sex, sampling year, sampling site, and MML location in a secured, web-based database called Type-Ned^26^. During an existing surveillance program on carbapenemase-producing *Enterobacterales* that began before this study, the RIVM had also received *P. aeruginosa* isolates from several MMLs, so isolates received in 2015 were also considered.

*P. aeruginosa* isolates submitted between January 1, 2015 to December 31, 2017 were characterized as follows: species level identification was confirmed using MALDI-TOF MS (Bruker Daltonik, Bremen, Germany), carbapenemase production was assessed using the CIM test^27^, and the detection of *bla*_VIM_, *bla*_IMP_, *bla*_KPC_, *bla*_OXA-48_, and *bla*_NDM_ genes was performed using multiplex PCR with primers and conditions as previously described^26,27^. CIM-positive *P. aeruginosa* isolates, and a subset of CIM-negative *P. aeruginosa* isolates matched by sampling year and MML, were subjected to sequencing.

### Whole-genome sequence analyses

NGS was performed using Illumina HiSeq 2500 (Illumina, San Diego, CA, USA), resulting in reads with 125 bases length. De novo assembly was performed using CLC Genomics Workbench v9.5.3 (Qiagen Bioinformatics, Aarhus, Denmark), and contig sequences with a minimum length of 500 bp and at least 30× average read coverage per contig were used for further analyses. QRDR analysis was performed using the sequence extraction tool in BioNumerics v7.6 (Applied Maths, Sint-Martens-Latem, Belgium), in which extracted sequences were translated and aligned to identify coding sequence changes. Sequence types and serotypes were inferred from NGS data in SeqSphere v3.5.0 (Ridom, Münster, Germany) as well as PAst software from the Center for Genomic Epidemiology^28^. Identification of wgMLST alleles was performed in SeqSphere using an in-house wgMLST scheme comprising 6117 core genes and 325 accessory genes based on the fully sequenced and annotated *P. aeruginosa* strain RIVM-EMC4982 (accession no. CP016955). Allelic distances between isolates were calculated using BioNumerics v7.6. Genes absent in the sequenced isolates were ignored and not counted as allelic differences.

For long-read sequencing, the Oxford Nanopore protocol SQK-LSK108 (https://community.nanoporetech.com) and the expansion kit for native barcoding EXP-NBD104 was used. DNA was repaired using FFPE and end-repair kits (New England BioLabs, Ipswich, MA, USA), followed by ligation of barcodes with bead cleanup using AMPure XP (Beckman Coulter, Brea, CA, USA) after each step. Barcoded isolates were pooled, and sequencing adapters were added by ligation. The final library was loaded onto a MinION flow cell (MIN-106 R9.4.1). After a 48-h sequence run, base calling and de-multiplexing was performed using Albacore 2.3.1, and a single FASTA file per isolate was extracted from the FAST5 files using Poretools 0.5.1 (https://www.github.com/arq5x/poretools)^29^. Illumina and Nanopore data were used in a hybrid assembly performed by Unicycler v0.4.4 (https://github.com/rrwick/Unicycler)^30^.

Antibiotic resistance gene profiles were generated by using the ResFinder program v3.2, and the database available from the Center for Genomic Epidemiology website (https://bitbucket.org/genomicepidemiology/resfinder/src/master; accessed 08-05-2020)^31^. For resistance gene identification, a 90% identity threshold and a minimum length of 60% were used as criteria.

### Ethical statement

The standard administrative procedure for carbapenemase-producing *Enterobacterales* was used to collect strains and demographic data^26^. Patient identifiers provided by MMLs were encrypted and then stored in the Type-Ned database, ensuring patient privacy in accordance with General Data Protection Regulation. Ethical approval was not required.

## Supplementary Information

Supplementary Information 1.

Supplementary Information 2.

Supplementary Information 3.

## Data Availability

Sequence data on the isolates in this paper have been deposited in the European Nucleotide Archive under study accession number PRJEB39528 (https://www.ebi.ac.uk/ena/browser/view/PRJEB39528).
