# Supplementary material for: National surveillance pilot study unveils a multicenter, clonal outbreak of VIM-2-producing Pseudomonas aeruginosa ST111 in the Netherlands between 2015 and 2017
Source: Sci Rep. 2021 Oct 25;11:21015. doi: 10.1038/s41598-021-00205-w (PMC8545960; doi:10.1038/s41598-021-00205-w)
Supplement: Supplementary file 1 — Supplementary Information 1. [file 41598_2021_205_MOESM1_ESM.docx]

National surveillance pilot study unveils a multicenter, clonal outbreak of VIM-2-producing *Pseudomonas aeruginosa* ST111 in the Netherlands between 2015-2017

Jannette Pirzadian^1^†, Marjolein C. Persoon^1,2^†, Juliëtte A. Severin^1^, Corné H. W. Klaassen^1^, Sabine C. de Greeff^3^, Marcel G. Mennen^3,4^, Annelot F. Schoffelen^3^, Cornelia C. H. Wielders^3^, Sandra Witteveen^5^, Marga van Santen-Verheuvel^5^, Leo M. Schouls^5^§, Margreet C. Vos^1*^§, and the Dutch CPE surveillance Study Group^6^

**Affiliations:**

^1^Department of Medical Microbiology and Infectious Diseases, Erasmus MC University Medical Center Rotterdam, Rotterdam, the Netherlands.

^2^Department of Medical Microbiology, University Medical Center Utrecht, Utrecht, the Netherlands.

^3^Center for Infectious Diseases, Epidemiology and Surveillance, Center for Infectious Disease Control, National Institute for Public Health and the Environment (RIVM), Bilthoven, the Netherlands.

^4^Center for Environmental Safety and Security, National Institute for Public Health and the Environment (RIVM), Bilthoven, the Netherlands.

^5^Center for Infectious Diseases Research, Diagnostics and Laboratory Surveillance, Center for Infectious Disease Control, National Institute for Public Health and the Environment (RIVM), Bilthoven, the Netherlands.

^6^Center for Infectious Disease Control, National Institute for Public Health and the Environment (RIVM), Bilthoven, the Netherlands. Representative: D.W. Notermans.

***Correspondence:** Prof. Dr. Margreet C. Vos, m.vos@erasmusmc.nl.

†These authors have contributed equally to this work and share first authorship.
§These authors have contributed equally to this work and share senior authorship.

**Supplementary Table S1.** Demographic data on patients from which 168 *P. aeruginosa* isolates were obtained and sequenced in this study. In total, 112 CIM-positive *P. aeruginosa* isolates and 56 CIM-negative *P. aeruginosa* isolates were sequenced.

|  | CIM-positive (%) | CIM-negative (%) |
| --- | --- | --- |
| Mean patient age in years [range] | 63 [0–92] | 64 [6–102] |
|  |  |  |
| Sex |  |  |
| Male | 74 (65%) | 31 (56%) |
| Female | 37 (33%) | 23 (42%) |
| Unknown | 2 (2%) | 1 (2%) |
|  |  |  |
| Isolate origin |  |  |
| Blood | 7 (6%) | 0 (0%) |
| Catheter entrance | 2 (2%) | 0 (0%) |
| Nose | 1 (0·9%) | 0 (0%) |
| Perineum | 8 (7%) | 4 (7%) |
| Pus | 4 (4%) | 0 (0%) |
| Rectum | 20 (18%) | 4 (7%) |
| Sputum | 18 (16%) | 12 (22%) |
| Throat | 5 (4%) | 2 (4%) |
| Urine | 25 (22%) | 15 (27%) |
| Wound | 7 (6%) | 3 (5%) |
| Other | 13 (12%) | 8 (15%) |
| Unknown | 3 (3%) | 7 (13%) |
|  |  |  |
| Isolation year |  |  |
| 2015 | 26 (23%) | 17 (31%) |
| 2016 | 27 (24%) | 20 (36%) |
| 2017 | 60 (53%) | 18 (33%) |
|  |  |  |
| Region (Dutch province)* |  |  |
| East (Overijssel) | 0 (0%) | 1 (2%) |
| North-West (Noord-Holland) | 27 (24%) | 13 (24%) |
| South-West (Zeeland) | 6 (5%) | 2 (4%) |
| South-West (Zuid-Holland) | 37 (33%) | 16 (29%) |
| Mid (Utrecht) | 9 (8%) | 3 (5%) |
| Mid (Gelderland) | 8 (7%) | 5 (9%) |
| South (Noord-Brabant) | 4 (4%) | 3 (5%) |
| South (Limburg) | 22 (19%) | 12 (22%) |

*The region where the MML that submitted the isolate was located, and not necessarily the region where the patient lived.

**Supplementary Figure S1.** Geographical distribution of *P. aeruginosa* isolates that were included in the study based on the province of the participating MML. Shown in red is the proportion of isolates that were CIM-positive, and in green is the proportion of isolates that were CIM-negative. In some provinces, the number of *P. aeruginosa* isolates submitted by MMLs was few to none. Figure was created by importing a screen capture into Adobe Illustrator (Adobe Creative Cloud 2020, www.adobe.com), and plotting the origin of isolates using the Type-Ned MRSA website (www.typened-mrsa.rivm.nl).

**
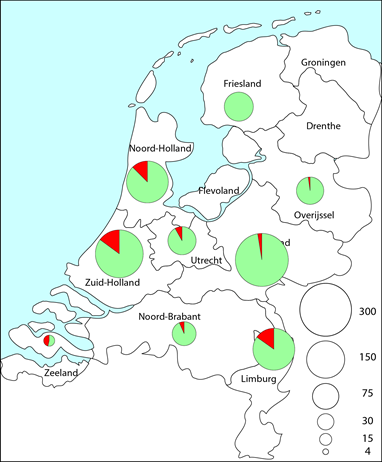
**
